# Distribution and mortality factors of cases with traumatic and nontraumatic brain damage treated in ICU

**DOI:** 10.1186/cc14612

**Published:** 2015-03-16

**Authors:** S Goksu Tomruk, N Bakan, G Karaören, E Akcay, S Keskin

**Affiliations:** 1 Umraniye Research and Training Hospital, Istanbul, Turkey

## Introduction

Cases of traumatic and nontraumatic brain damage have high rates of morbidity and mortality. In this study of cases being treated in the ICU for a diagnosis of brain damage, it was aimed to evaluate the relationship between mortality and the distribution of reason for and resulting type of brain damage and to determine other factors affecting mortality.

## Methods

After local ethics committee approval, a total of 1,004 patients (2012, *n *= 492; 2013, *n *= 454; 2014, *n *= 58) treated in the ICU in a 2-year period were reviewed. This study included for evaluation 135 patients determined with traumatic or nontraumatic brain damage, with a more than 24-hour stay in the ICU. Reasons for brain damage were determined as brain damage associated with head trauma (Group HT), head trauma accompanying general body trauma (Group HT + GBT) and spontaneous haemorrhage (Group SH). The type of brain damage was defined from the radiological diagnosis (CT and/or MRI) as subarachnoid haemorrhage, intracranial haemorrhage (ICH), subdural haematoma (SDH), epidural haematoma (EDH), skull fracture, brain contusion or a combination of these (COM). Operations were evaluated as performed by the brain surgery department.

## Results

The patients comprised 73.3% males and 26.7% females with a mean age of 40.58 ± 24.14 years (range, 1 to 87 years), mean APACHE II score of 19.07 ± 10.19 (range, 2 to 41), mean GCS of 9.17 ± 4.42 (range, 3 to 15) and 68.1% of whom were discharged and 31.9% were exitus. When the causes of brain damage were evaluated according to the type, the most frequently seen in the HT and HT + GBT groups were COM (37.3%, 42.9%), followed by EDH (13.6%, 28.6%). In the SH group, the most common reason was ICH (43.9%) followed by SDH (24.4%). Directly proportionally, only an increase in APACHE II score increased the mortality risk 1.3-fold (logistic regression analyses, coefficient 0.658) but the duration of intubation and ICH ratio was statistically significantly high and GCS was low in the exitus cases, and rates of EDH were determined as high in discharged cases compared with exitus (*P *< 0.01). No statistical difference was determined in mortality in terms of age, gender, duration in ICU, surgical treatment or not, or reasons for brain damage (*P >*0.05).

## Conclusion

There is considerable variation in causes of head injury. From this retrospective study it can be suggested that mortality of neurological/neurosurgical patients, regardless of management method, depends on APACHE II, arrival GCS, number of ventilator-days and type of brain damage.

